# Confinement-Tunable Spatial Distribution of Physisorbed Hydrogen in Defective Carbon Nanotube Bundles

**DOI:** 10.3390/e28040415

**Published:** 2026-04-07

**Authors:** Shuming Yang, Kun Qiu, Gang Sun, Huaze Shen

**Affiliations:** 1 Center for Advanced Quantum Studies, School of Physics and Astronomy, Beijing Normal University, Beijing 100875, China; yangshuming23@mails.ucas.ac.cn (S.Y.); kunqiu@mail.bnu.edu.cn (K.Q.); 2 Beijing National Laboratory for Condensed Matter Physics, Institute of Physics, Chinese Academy of Sciences, Beijing 100190, China; 3 School of Physical Sciences, University of Chinese Academy of Sciences, Beijing 100049, China; 4 School of Electronic and Information Engineering, Chongqing University of Arts and Sciences, Chongqing 402160, China

**Keywords:** spatial confinement, single-walled carbon nanotube bundles, hydrogen physisorption, gas separation

## Abstract

Spatial confinement strongly affects matter by altering structural stability, relaxation times, and equilibrium properties. Interest in hydrogen storage within carbon nanotube bundles has grown because it addresses practical energy needs while revealing rich confined-fluid physics. Understanding how geometry and defects influence hydrogen structure and dynamics is essential to the development of effective storage materials. Here, we investigate how confinement in single-walled carbon nanotube (SWCNT) bundles with vacancies alters the spatial distribution and phase behavior of physisorbed hydrogen. At low temperature, hydrogen forms solid-like, cylindrical layered structures both inside and outside the tubes. Raising the temperature broadens these layers and produces a liquid-like arrangement within the confined regions. This confined solid-to-liquid crossover controls storage capacity and release behavior and can be tuned by temperature, confinement dimensions, and vacancy defects.

## 1. Introduction

Spatial confinement profoundly alters the behavior of matter [1,2,3]. Restricting particles to nanoscale regions changes available energy states, enhances interface effects, and modifies transport pathways [4,5,6]. These changes can produce altered density of states, dimensional crossovers, and size-dependent phase behavior, all of which are absent in bulk systems [7,8]. Understanding how geometry, boundary conditions, and defects control structure and dynamics in confined environments is therefore central both to fundamental physics and to the design of functional nanoscale materials.

Carbon nanotubes (CNTs) are an archetypal confined geometry. Their hollow, high-aspect-ratio interiors, well-defined diameters, and tunable surface chemistry provide an ideal platform to study low-dimensional fluids and host–guest interactions [9,10,11]. In bundles, additional confinement arises from interstitial channels and groove sites, producing a variety of adsorption environments with distinct energetics and dimensionalities [11,12]. At the same time, nanotube systems combine excellent mechanical and chemical stability with a high specific surface area, making them attractive candidates for gas adsorption applications [9,10].

Hydrogen is an ideal fuel for the future because of its high internal energy, great abundance, and environmentally friendly reaction products [13,14,15,16,17,18,19,20]. It has the potential to be applied in various scenarios, such as producing electricity or serving as fuel for automobiles [13,17]. However, thus far, hydrogen has remained underutilized, and one of the most critical challenges is the lack of efficient hydrogen storage methods [14,15,16,17,18,19,20,21]. In recent years, scientists have proposed using many kinds of materials and methods for hydrogen storage [21,22,23,24,25,26,27,28,29,30,31,32,33], among which hydrogen physisorption on SWCNTs is believed to be a good choice [33,34,35,36,37,38,39]. A lot of studies focus on hydrogen adsorption on perfect SWCNTs. However, defects can always be found on the walls of SWCNTs in practice [40,41,42,43,44,45,46,47,48,49]. Therefore, in our previous work [1], we investigated the physisorption of H_2_ inside an isolated SWCNT with vacant defects. We found that H_2_ can be stored inside the nanotube through these vacancies, and that increasing the number of defects can enhance adsorption efficiency. Additionally, SWCNTs tend to assemble into bundles arranged in a hexagonal pattern [37,38,50,51,52,53,54,55,56,57,58,59,60]. Studies have shown that gas adsorption behavior varies depending on the site within these bundles [38,52,54,55,57,58]. For example, studies have explored the adsorption of noble gases (e.g., Ne [52], Xe [52,54], Kr [55]) as well as H_2_ [38], N_2_ [57,58], O_2_ [58], and CH_4_ [55] at different sites within SWCNT bundles. Yin et al. [57] studied the physisorption of N_2_ in SWCNT bundles and found that SWCNT bundles exhibit great potential for achieving high adsorptive capacities compared to isolated nanotubes. Considering these findings, both the introduction of vacant defects [1] and the formation of SWCNT bundled structures [57] are promising strategies for enhancing gas storage efficiency in SWCNTs.

In this work, we employ molecular dynamics (MD) simulations to systematically investigate the physisorption of H_2_ confined in SWCNT bundles featuring vacant defects on their walls. We focus on how spatial confinement and external parameters (e.g., temperature and pressure) affect the structure of adsorbed hydrogen. Additionally, we explore the selective adsorption of H_2_ from a H_2_/N_2_ gas mixture by adjusting the inter-nanotube distance. Our findings indicate that SWCNT bundles with vacant defects can effectively select H_2_ from a gas mixture containing H_2_ and N_2_. Moreover, increasing the number of vacant defects on the wall enhances hydrogen adsorption efficiency.

This paper is organized as follows: Section 2 describes the simulation methods, Section 3 presents our main results—covering the spatial distribution and phase behavior of hydrogen molecules in SWCNT bundles, the influence of external parameters and bundle characteristics on H_2_ adsorption, as well as the selective separation of H_2_ from H_2_/N_2_ mixtures—and Section 4 provides a summary.

## 2. Methods

**Figure 1:**
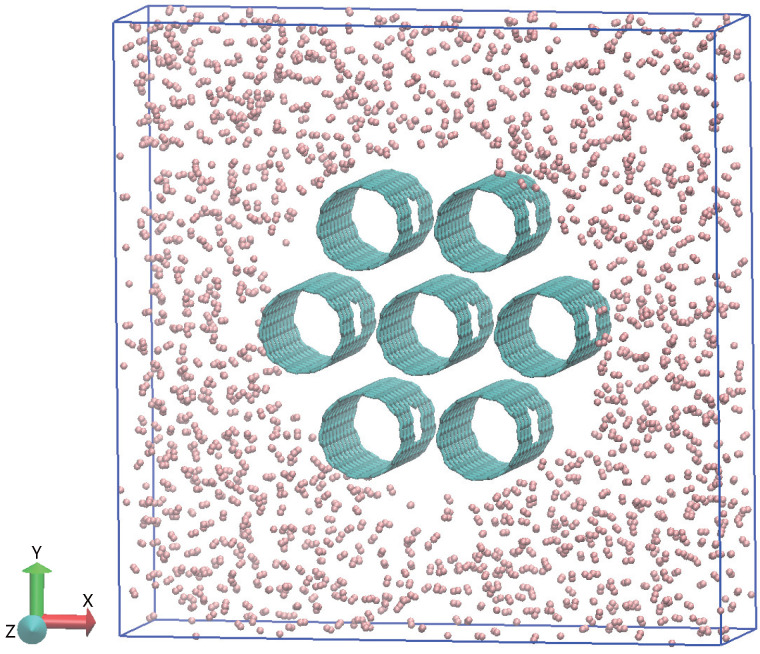
Schematic illustration of the studied system, which consists of SWCNT bundles and hydrogen molecules. The SWCNT bundles (colored cyan) are packed in a hexagonal array. Each SWCNT has a vacant defect on its wall. The hydrogen molecules (colored pink) are initially distributed outside the SWCNT bundles.

We perform molecular dynamics (MD) simulations on a system consisting of hydrogen molecules and SWCNT bundles with vacant defects. In our system, the SWCNT bundles consist of seven isolated SWCNTs—each with vacant defects on its wall—packed together in a hexagonal array, as shown in Figure 1. The inter-nanotube distance between adjacent nanotubes in the SWCNT bundles varies from 3 to 9 Å. Each nanotube adopts the armchair configuration and has a length of 24.6 Å. The diameter of each SWCNT is determined by controlling its chiral index, which varies from (6, 6) (with a diameter of 8.14 Å) to (12, 12) (with a diameter of 16.27 Å). The vacant defect is obtained by removing N_*V*_ carbon atoms from the wall of each SWCNT, where N_*V*_ is defined as the size of the vacant defect.

In our study, the number of hydrogen molecules in the simulation box varies from 2000 to 8000, specifically to investigate the effect of pressure on H_2_ physisorption. For the selective adsorption of H_2_ from a H_2_/N_2_ gas mixture, the system consists of 1500 H_2_ molecules and 1500 N_2_ molecules. To explore the effect of multiple defects on hydrogen adsorption, multiple defects are created on the wall of each SWCNT in the bundles.

There are two different types of interactions between atoms in the simulation box, namely intra-molecular and inter-molecular interactions. Intra-molecular interactions—such as the covalent bonds within H_2_ and N_2_ molecules—are described by a harmonic spring model [1]. Inter-molecular interactions are described by the Lennard–Jones potential:

$$U\left(r_{ij}\right)=4\varepsilon \left[{\left(\frac{\sigma }{r_{ij}}\right)}^{12}-{\left(\frac{\sigma }{r_{ij}}\right)}^{6}\right]$$

where σ denotes the size parameter and ε denotes the energy parameter. We adopt the same parameters for the intra- and inter-molecular interactions as in Ref. [1]. The SWCNT bundles are kept fixed throughout simulations, and thus the interactions between carbon atoms can be neglected.

In this study, the simulation cell is a cube with the following dimensions: Lx = 90.5 Å, Ly = 90.5 Å, Lz = 24.6 Å. All MD simulations are performed in LAMMPS [61] (version: 22 Jul 2025) using the NVT ensemble with periodic boundary conditions applied in all three directions. The equations of motion are integrated using the velocity Verlet algorithm with a time step of 1 fs. The temperature is controlled with a Nose–Hoover thermostat with a damping parameter of 100 fs.

**Figure 2:**
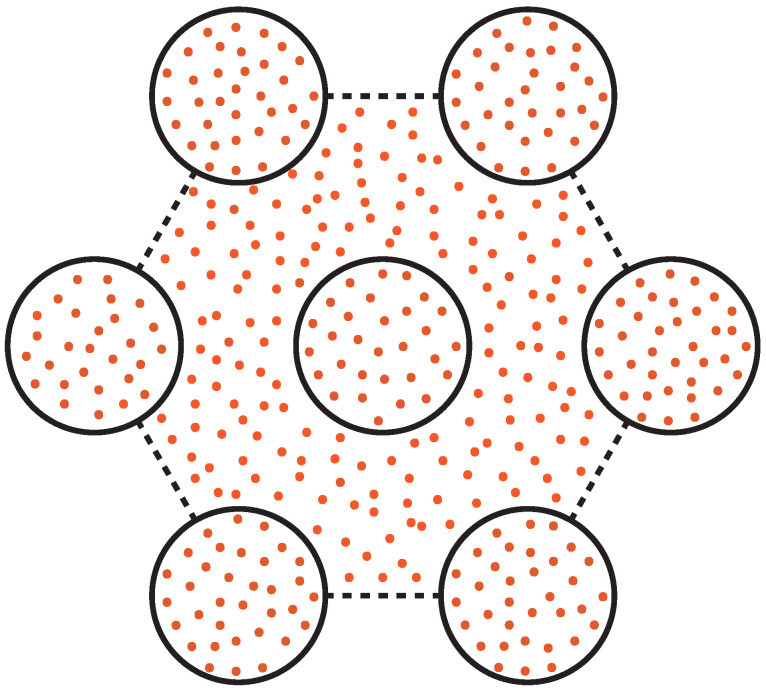
Adsorption of H_2_ in SWCNT bundles in a hexagonal array. The adsorbed H_2_ molecules include two parts: inside each SWCNT tube and in the interstitial region between SWCNTs. The dots in orange indicate the H_2_ molecules. Here, we use a geometrical method to define the boundary of the interstitial region, which is defined as the dashed line connecting the centers of two outermost adjacent SWCNT tubes.

To quantitatively evaluate the adsorption efficiency, we classify hydrogen molecules as physisorbed on the basis of their geometric positions [57,62,63,64]. As shown in Figure 2, a hydrogen molecule is considered adsorbed if it is located in the confined regions (i.e., inside each nanotube or within interstitial spaces between nanotubes). The boundary of the interstitial region is defined as the straight lines connecting the centers of each pair of outermost adjacent SWCNTs. Based on this definition, we can quantify the number of hydrogen molecules adsorbed onto the SWCNT bundles. For comparison with the hydrogen adsorption efficiency of isolated SWCNT, the average number of hydrogen molecules adsorbed within each SWCNT of the bundles is divided by 7, and the value is denoted as N_*adsorb*_.

## 3. Results

### 3.1. Spatial Distribution and Phase Behavior of Hydrogen Molecules in SWCNT Bundles

**Figure 3:**
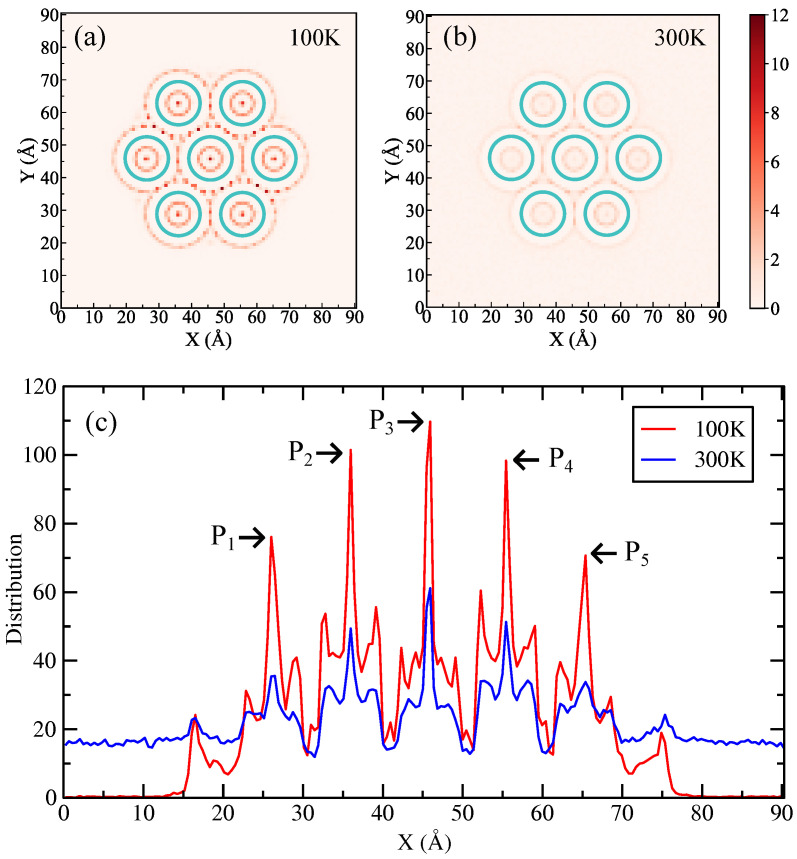
Spatial distribution of hydrogen molecules in SWCNT bundles. Panels (**a**) and (**b**) show the two-dimensional density distribution of hydrogen molecules projected onto the x-y plane in SWCNT bundles at 100 K and 300 K, respectively. The cyan circles denote the SWCNT bundles. The color scale from white to red indicates an increase in atomic density. Panel (**c**) shows the one-dimensional density distribution of hydrogen molecules in SWCNT bundles along the x-axis. The red line and blue line correspond to 100 K and 300 K, respectively.

We begin by analyzing the spatial distribution and phase behavior of H_2_ molecules confined in defective SWCNT bundles. Each simulation contains 1000 H_2_ molecules equilibrated in the NVT ensemble at T = 100 K and T = 300 K. After equilibration, we extract the two-dimensional density projected onto the xy plane (Figure 3a for 100 K, Figure 3b for 300 K) and the one-dimensional density along the x-axis (Figure 3c) to quantify ordering and adsorption sites.

At 100 K, the density maps show pronounced layering of H_2_ both inside individual nanotubes and in the interstitial regions between adjacent nanotubes. The x-projected density is essentially zero outside the bundle (x < 10 Å and x > 80 Å) and exhibits five well-resolved peaks ($P_{1}$–$P_{5}$), whose positions coincide with the centers of the nanotubes in the simulated bundle geometry ($P_{1}\approx 26$ Å, $P_{2}\approx 36$ Å, $P_{3}\approx 46$ Å, $P_{4}\approx 56$ Å, $P_{5}\approx 66$ Å). The sharpness and regular spacing of these peaks indicate a strong positional correlation and suppressed translational motion of H_2_ molecules—a nearly “solid-like” arrangement. Physically, this ordered arrangement arises from the combined effects of the attractive physisorption potential of the nanotube walls and the geometric confinement imposed by tube curvature and inter-tube spacing: minima in the potential energy landscape favor occupation at tube centers and stable interstitial sites, while low thermal energy at 100 K limits hopping between adjacent energy minima.

Increasing the temperature to 300 K produces a markedly different distribution. The two-dimensional density broadens and the x-projected peaks are reduced in amplitude and broadened; simultaneously, the density outside the bundle region (x > 10 Å and x < 80 Å) increases. These changes reflect the partial desorption and enhanced mobility of H_2_: the thermal energy overcomes shallow adsorption potential wells and interstitial binding interactions, thus increasing the escape rate of H_2_ molecules from confined sites and facilitating their diffusion along and away from the bundle. The structural transition of the density profile from sharp, localized peaks to a more continuous distribution is consistent with a loss of long-range translational order and a crossover toward “fluid-like” behavior.

The observed temperature-dependent behavior can be understood from the perspectives of thermodynamics and kinetics. At low temperature, the system minimizes potential energy by occupying deep adsorption energy minima, yielding a high local density and a reduced configurational entropy; the resulting state is energy-dominated and spatially ordered. At higher temperature, the entropic contribution and thermal activation energy become comparable to the adsorption energies of binding sites, thus favoring molecular delocalization, increased residence-time heterogeneity, and net desorption. Defects in the SWCNT bundles modify the local potential landscape: they can create deeper binding sites that retain H_2_ at higher temperatures, or open pathways that facilitate diffusion between tubes and into the external environment. Thus, the structural characteristics of defects influence both the onset temperature of delocalization and the relative populations in intra-tube versus interstitial and external sites.

From a practical perspective, these results imply that physisorption-based storage in SWCNT bundles exhibits strong temperature sensitivity: low-temperature conditions favor dense, ordered loading, while modest temperature increases significantly reduce confinement and increase release. For applications, this suggests a trade-off between storage density (favored by strong confinement and low temperature) and reversible delivery (favored by moderate binding allowing thermal release). Future work should quantify adsorption energies for distinct sites, measure diffusion coefficients as a function of temperature, and examine how varying defect type and bundle packing alter the energy landscape and kinetics. These additional data would enable predictive modeling of uptake, retention, and release behavior under experimentally relevant thermal cycles.

### 3.2. Effects of Temperature and Pressure on Physisorption

We then investigate the effect of external parameters—such as temperature and pressure—on hydrogen physisorption in SWCNT bundles with vacant defects. The SWCNT used here is the (10, 10) armchair style and it has a diameter of 13.56 Å. The size of vacant defect is set to N_*V*_ = 9. The inter-nanotube distance between adjacent SWCNTs is maintained as 6 Å. To study the temperature effect, we introduce 3000 H_2_ molecules into the system while maintaining a constant H_2_ density and then change the temperature of the system from 80 K to 300 K.

**Figure 4:**
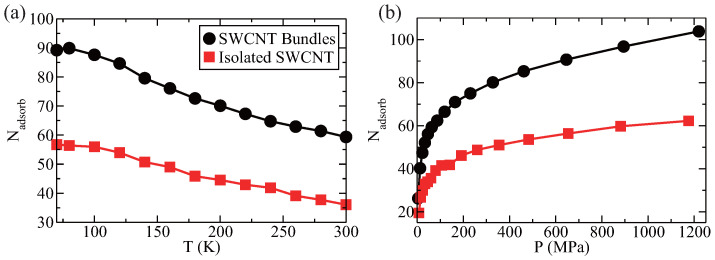
Effects of temperature (**a**) and pressure (**b**) on hydrogen adsorption in SWCNT bundles and isolated SWCNT (for comparison). Similar to that in isolated SWCNT [1], the adsorption efficiency of SWCNT bundles decreases as temperature increases and increases as pressure increases—a trend consistent with isolated SWCNT. However, SWCNT bundles exhibit significantly higher hydrogen adsorption efficiency than isolated SWCNTs.

As shown in Figure 4a, the number of hydrogen molecules adsorbed in both SWCNT bundles and the single SWCNT decreases as the temperature increases from 80 K to 300 K. This indicates that the adscorption efficiency declines with rising temperature. Comparing the two, SWCNT bundles exhibit higher adsorption capacity than isolated SWCNTs across the temperature range. We examine the effect of pressure on hydrogen storage in SWCNT bundles at a fixed temperature of 300 K. By adjusting the hydrogen density, the pressure varies from 4 MPa to 1219 MPa. Figure 4b illustrates how pressure influences hydrogen storage in both SWCNT bundles and isolated SWCNTs. It is found that increasing pressure enhances adsorption efficiency, eventually reaching saturation at higher pressures. Therefore, on average, more hydrogen molecules are adsorbed by each SWCNT in the bundles than that in isolated SWCNT. This indicates that both temperature and pressure are effective control parameters for hydrogen storage in SWCNTs bundles. The SWCNT bundling significantly improves the hydrogen storage capacity.

### 3.3. The Effect of Parameters of SWCNT Bundles on Physisorption

**Figure 5:**
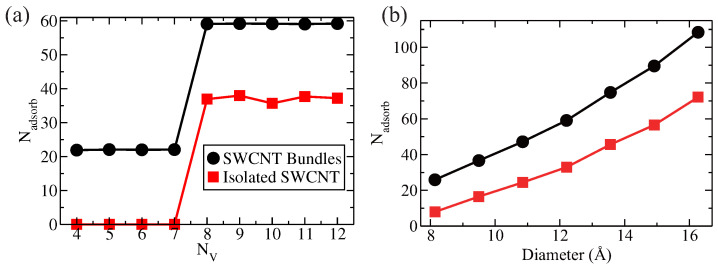
Effect of SWCNT’s vacant defect size (**a**) and diameter (**b**) on H_2_ adsorption in SWCNT bundles and isolated SWCNT. (**a**) A critical defect size ($N_{V}$ = 8) exists, below which no H_2_ molecules can be adsorbed inside the nanotube. (**b**) In both SWCNT bundles and isolated SWCNT systems, the hydrogen storage capacity increases with increasing SWCNT diameter.

For the SWCNT bundles with vacant defects, three main factors influence hydrogen adsorption: defect size, nanotube diameter, and the inter-nanotube distance. In the case of a single isolated SWCNT, previous studies have identified a critical defect size, below which hydrogen physisorption through the vacancy is not feasible [1]. This is because hydrogen molecules must overcome a high energy barrier to enter the nanotube via small vacancies. This critical defect size, denoted as $N_{V}^{C}$, is approximately eight. As a comparison study, we examine how defect size affects hydrogen storage in SWCNT bundles under consistent conditions: 3000 hydrogen molecules, temperature 300 K, armchair (10, 10) nanotubes with a diameter of 13.56 Å, and an inter-tube distance of 6 Å. When varying the defect size N_*V*_ from 4 to 12 (see Figure 5a), we observe that a similar critical defect size, N_*V*_ = 8, exists for bundles as well—below this value, hydrogen molecules cannot be adsorbed inside the nanotubes. These findings align with previous results for isolated SWCNTs.

Next, we investigate how the diameter of SWCNTs influences hydrogen adsorption. Here we fix the temperature at 300 K, with a defect size N_*V*_ = 9, inter-nanotube distance $d_{tube}$ = 6 Å, and a total of 3000 hydrogen molecules. We vary the nanotube chirality from (6, 6) to (12, 12), corresponding to diameters ranging from 8.14 Å to 16 Å. The results, presented in Figure 5, show that hydrogen adsorption capacity increases as the nanotube diameter grows. SWCNT bundles consistently exhibit higher hydrogen storage capacity than isolated SWCNTs.

**Figure 6:**
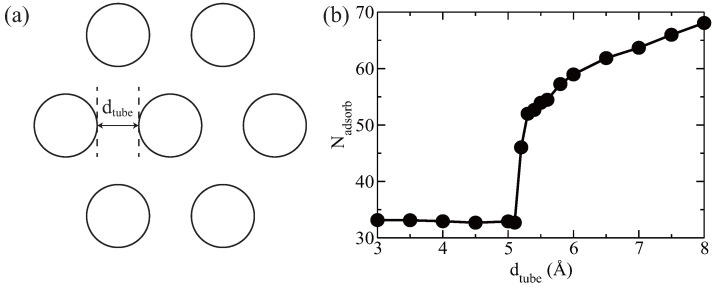
The effect of inter-nanotube distance $d_{tube}$ on the hydrogen adsorption in SWCNT bundles. (**a**) Illustration of the inter-nanotube distance of the SWCNT bundles $d_{tube}$. (**b**) Adsorption of H_2_ as a function of $d_{tube}$. For $d_{tube}$ < 5.1 Å, H_2_ cannot enter the interstitial region and can only be adsorbed inside the SWCNT in the bundles. For $d_{tube}$ > 5.1 Å, H_2_ can enter the interstitial region, then the SWCNT bundles can adsorb more H_2_.

Unlike isolated SWCNTs, bundles introduce an additional parameter—namely, the inter-nanotube distance—which can influence hydrogen adsorption efficiency. To examine this effect, we vary the inter-nanotube distance $d_{tube}$ from 3 Å to 8 Å with the defect size N_*V*_ = 9 and a nanotube diameter of 13.56 Å at T = 300 K (see Figure 6a). Our results indicate a critical inter-nanotube distance of approximately 5.1 Å (see Figure 6b). Below this value, hydrogen molecules are only adsorbed inside individual nanotubes, as they cannot enter the space between neighboring tubes. When the distance exceeds 5.1 Å, hydrogen molecules can access the interstitial regions, resulting in increased storage capacity. Notably, there is a sharp rise in the number of adsorbed hydrogen molecules when the inter-nanotube distance is between 5.1 Å and 5.3 Å. Beyond 5.3 Å, the interstitial space is sufficiently large to be fully occupied, and further increases in the inter-tube distance lead to a proportional rise in hydrogen storage, corresponding to the expanding volume of the interstitial region.

### 3.4. Comparison of Adsorption Efficiency Between Single and Multiple Defects

Previous research [1] shows that the total number of hydrogen molecules adsorbed inside an isolated SWCNT is independent of the number of vacancy defects on its wall. This suggests that increasing the number of vacant defects in SWCNT bundles could enhance hydrogen storage capacity. To explore this, we compare hydrogen adsorption efficiency between isolated SWCNTs and SWCNT bundles with either a single defect or multiple defects per nanotube. Here, the single defect case indicates that each SWCNT in bundles has only one vacancy with N_*V*_ = 9. For the multiple-defects, we consider an extreme case, e.g., each SWCNT has 15 holes on the SWCNT among which 12 holes are of size $N_{V}=6$ and three are of size $N_{V}=9$, which is detailed in the previous work [1]. In the extreme case, adding one more defect in the SWCNT leads to overlapping with the existing defects.

**Figure 7:**
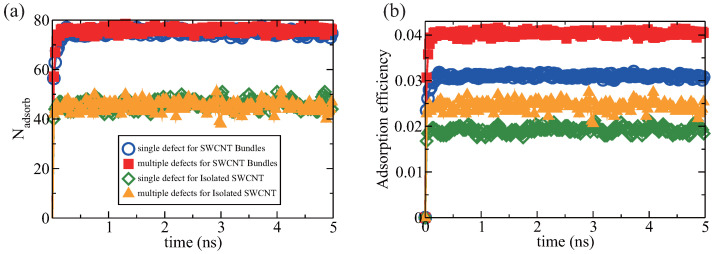
Comparison of H_2_ adsorption efficiency in isolated SWCNT and SWCNT bundles with single and multiple vacant defects. (**a**) The number of adsorbed hydrogen molecules in both isolated SWCNT and SWCNT bundles as a function of time. (**b**) The hydrogen adsorption efficiency (defined as the mass of adsorbed hydrogen molecules divided by the sum of the mass of adsorbed hydrogen molecules and the mass of SWCNTs) in four cases: isolated SWCNT with a single defect, isolated SWCNT with multiple defects, SWCNT bundles with single defect on each SWCNT’s wall, and SWCNT bundles with multiple defects on each SWCNT’s wall.

As shown in Figure 7a, for both isolated SWCNTs and SWCNT bundles, the number of adsorbed H_2_ molecules is nearly identical under both single-defect and multiple-defect conditions, demonstrating that the number of vacant defects exerts a negligible effect on total hydrogen uptake. However, SWCNT bundles generally tend to adsorb more hydrogen molecules than isolated SWCNTs. Hydrogen storage efficiency is quantified as the mass ratio of adsorbed H_2_ to the combined mass of adsorbed H_2_ and the SWCNTs. As shown in Figure 7b, this efficiency varies among the four cases. For both the single- and multiple-defect situations, isolated SWCNTs adsorb similar amounts of hydrogen, but the multiple-defect SWCNT has fewer carbon atoms and its weight is reduced by about 24.8% compared to that of the perfect SWCNT. Consequently, the storage efficiencies of multiple-defect isolated SWCNT and SWCNT bundles are 2.44%±0.11 wt.% and 4.04%±0.04 wt.%, respectively. The SWCNT bundles with multiple defects exhibit the highest hydrogen storage efficiency. Notably, bundles with multiple defects show the highest storage efficiency; approximately twice that of single-vacancy nanotubes. These results support the idea that increasing defect density and bundling both enhance hydrogen storage performance.

### 3.5. Selection of Hydrogen Molecules from a Gas Mixture

In practical applications, hydrogen storage often involves separating H_2_ from polluted gas mixtures, such as H_2_/N_2_. Therefore, the ability of SWCNT bundles to selectively adsorb hydrogen from such mixtures is of significant importance. Building on our previous study of isolated SWCNT [1], we now investigate the performance of SWCNT bundles in selecting H_2_ from an H_2_/N_2_ gas mixture.

**Figure 8:**
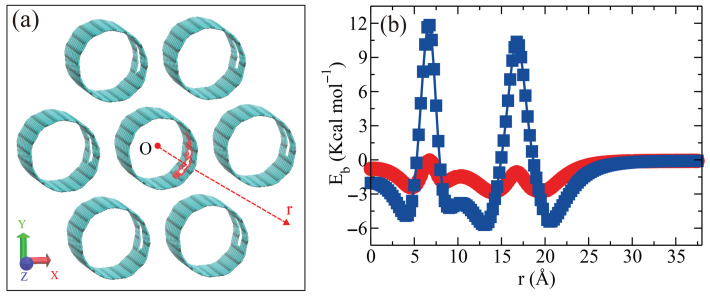

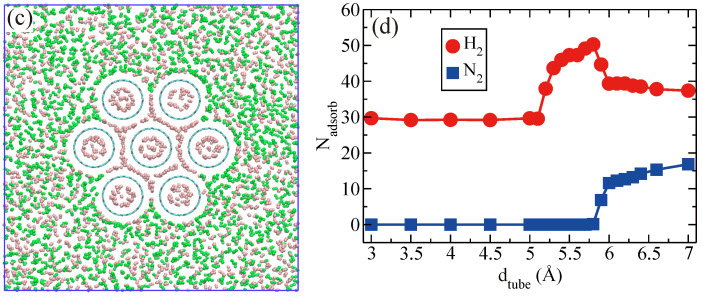
Selectivity of SWCNT bundles for H_2_ over N_2_. (**a**) Reaction coordinate used to compute adsorption energies: a straight path beginning at the center of the central nanotube, passing through a vacancy on its wall, traversing the midpoint between two adjacent outermost nanotubes, and finally extending to the exterior of the SWCNT bundles. The SWCNT bundles are shown in cyan, except that the carbon atoms surrounding the vacancy on the wall of the central nanotube are shown in red. (**b**) Comparison of adsorption energies $E_{b}$ for H_2_ and N_2_ along the reaction coordinate shown in (**a**). (**c**) Snapshot illustrating preferential uptake of H_2_ (pink) from an H_2_/N_2_ (green) mixture in SWCNT bundles (cyan circles). (**d**) Number of adsorbed H_2_ and N_2_ molecules versus inter-nanotube distance $d_{tube}$ for vacancy size $N_{V}$ = 9.

To access the confined regions of the SWCNT bundle, a gas molecule (H_2_ or N_2_) must pass either through a vacancy in a tube wall or through the interstitial channel between two adjacent outermost nanotubes, overcoming an associated energy barrier. To quantify the difference between H_2_ and N_2_, we compute the adsorption energy of a gas molecule (H_2_ or N_2_) along the reaction coordinate shown in Figure 8a. The vacant defect size and the inter-nanotube distance are set to N_*V*_ = 9 and $d_{tube}$ = 5.8 Å, respectively. The reaction coordinate is a straight line from the center of the central tube, through the vacancy, to the midpoint between two outermost tubes. The adsorption energy profiles (Figure 8b) exhibit two characteristic peaks for both species. The first peak, near 6.75 Å, corresponds to the energy barrier associated with the vacant defect and depends on N_*V*_. The second peak, near 16.75 Å, corresponds to the energy barrier associated with the interstitial channel between two adjacent outermost nanotubes and depends on $d_{tube}$. Adsorption of gas molecules into the SWCNT bundles is controlled by the energy barriers at 6.75 Å and 16.75 Å (see Figure 8b), respectively. The energy barriers for N_2_ at 6.75 Å and 16.75 Å are much higher than the corresponding ones for H_2_. N_2_ cannot enter the confined regions of the SWCNT bundles (i.e., the interior of each nanotube or the interstitial regions between nanotubes) because of the higher energy barriers, whereas H_2_ can easily access these regions. This difference in energy barrier heights—that is, the screening effect of the vacant defect and the inter-nanotube channel–accounts for the selective adsorption of H_2_ over N_2_ in the SWCNT bundles. Figure 8c presents a snapshot to illustrate the selective adsorption of H_2_ over N_2_ in the SWCNT bundles. To determine the optimal inter-nanotube distance $d_{tube}$, we systematically vary $d_{tube}$ and quantify the number of H_2_ and N_2_ molecules adsorbed in the SWCNT bundles. Figure 8d shows the number of adsorbed H_2_ and N_2_ molecules as a function of $d_{tube}$. For $d_{tube}$ < 5.1 Å, neither H_2_ nor N_2_ molecules can occupy the interstitial sites of the SWCNT bundles. However, with the vacant defect size N_*V*_ = 9, H_2_ molecules can still enter the interiors of outermost tubes whose defects face outward, but N_2_ cannot due to the higher energy barrier at the vacant defect. In the range 5.1–5.8 Å, H_2_ can access the interstitial regions while N_2_ remains excluded. Above 5.9 Å, both gases can enter the interstitial space. Therefore, the spacing that maximizes selectivity for H_2_ over N_2_ is approximately 5.8 Å. Compared with our previous work [1], this study identifies the inter-nanotube distance $d_{tube}$ as an additional control parameter for gas separation.

## 4. Summary

To summarize, we examine the physisorption of hydrogen molecules on SWCNT bundles with vacant defects and compare their storage performance with that of the isolated SWCNT. Our systematic study evaluates how both external factors (i.e., temperature and pressure) and intrinsic properties of the SWCNT bundles (i.e., nanotube diameter, vacancy defect size, and inter-nanotube distance) regulate the hydrogen adsorption efficiency. We find that at low temperatures, H_2_ adopts ordered, layered (solid-like) arrangements inside tubes and in interstitial sites. Increasing temperature broadens these layers, promotes desorption, and produces a more fluid-like distribution. Thus, temperature strongly controls uptake, mobility, and release. Raising pressure at 300 K increases adsorption and approaches saturation at high pressures. Bundled SWCNTs adsorb more H_2_ per tube than isolated SWCNTs over the entire investigated pressure range. For pure H_2_ adsorption, a critical inter-nanotube distance of approximately 5.1 Å exists in the SWCNT bundle system. Below 5.1 Å, H_2_ molecules cannot enter the interstitial regions between adjacent nanotubes and can only be adsorbed in the interior of each outermost nanotube; beyond 5.1 Å, H_2_ molecules can enter the interstitial regions between adjacent nanotubes and the interior of the central nanotube. For the selective adsorption of H_2_ from an H_2_/N_2_ gas mixture, the optimal inter-nanotube distance $d_{tube}$ is approximately 5.8 Å; at this spacing, H_2_ can enter the interstitial regions whereas N_2_ cannot. The total H_2_ uptake of the system is relatively insensitive to defect number, while the defect-induced mass reduction effectively enhances gravimetric storage efficiency. Multiple-defect bundles achieve the highest measured efficiencies, 4.04±0.04 wt.%, compared with 2.44±0.11 wt.% for multiple-defect isolated SWCNT. Overall, bundling of SWCNTs and increased vacant defect density jointly improve practical hydrogen storage performance metrics. Adsorption energy profiles along the reaction coordinate show two negative barriers for H_2_ but two positive barriers for N_2_ at $d_{tube}=5.8$ Å and N_*V*_ = 9, explaining the size-dependent selective adsorption of H_2_ in the SWCNT bundles.

Bundling of SWCNTs and controlled defects enhance H_2_ uptake and gravimetric efficiency, while inter-tube spacing offers an effective geometric handle to balance capacity and selectivity. Practical physisorption storage will require optimizing confinement geometry, defect engineering, and operating temperature and pressure to trade off between dense H_2_ loading and reversible H_2_ release. Future work should quantify site-specific binding energies, measure temperature-dependent diffusion coefficients, and map how specific defect types and bundle packings modify the adsorption energy landscape to support predictive design for storage and separation applications.

## Data Availability

All data generated or analyzed during this study are included in this published article.
